# Understanding the Link between Social Organization and Crime in Rural Communities

**Published:** 2015

**Authors:** Sarah M. Chilenski, Amy K. Syvertsen, Mark T. Greenberg

**Affiliations:** Prevention Research Center, Pennsylvania State University, State College, PA, United States; Search Institute, Minneapolis, MN; Prevention Research Center, Pennsylvania State University, State College, PA, United States

**Keywords:** rural, crime, social disorganization, collective efficacy, social trust, mediation

## Abstract

Rural communities make up much of America's heartland, yet we know little about their social organization, and how elements of their social organization relate to crime rates. The current study sought to remedy this gap by examining the associations between two measures of social organization – collective efficacy and social trust – with a number of structural community characteristics, local crime rates, and perceptions of safety in a sample of 27 rural and small town communities in two states. Measures of collective efficacy, social trust, and perceived safety, were gathered from key community members in 2006; other measures were drawn from the 2000 Census and FBI Uniform Crime Reporting system. A series of competing hypotheses were tested to examine the relative importance of social trust and collective efficacy in predicting local crime rates. Results do not support the full generalization of the social disorganization model. Correlational analyses showed that neither collective efficacy nor social trust had a direct association with community crime, nor did they mediate the associations between community structural characteristics and crime. However, perceived safety mediated the association between community crime and both measures of social organization. Analyses suggest that social trust may be more important than collective efficacy when understanding the effect of crime on a community's culture in rural areas. Understanding these associations in rural settings can aid decision makers in shaping policies to reduce crime and juvenile delinquency.

## 1.0 Introduction: Understanding the Link between Social Organization and Crime in Rural Communities

The social organization of communities has been shown to be an important factor in understanding multiple aspects of the social, emotional, and behavioral health of community residents and the community at large in largely urban and mixed urban/rural settings (Bjornstrom, 2011c; Dassopoulos & Monnat, 2011; Kawachi, Kennedy, & Wilkinson, 1999; Leventhal & Brooks-Gunn, 2000; Osgood, 2000; R. Putnam, 2000; R. D. Putnam, 1995; Sampson, 1997; Shaw & McKay, 1999). However, more research is needed with rural samples in order to better understand how social organization operates in rural and small town areas. Understanding these associations in rural settings can aid decision makers in shaping policies to improve the quality of life in these areas. Consequently, the current study uses a series of competing hypotheses in order to better understand how the social organization of rural and small town communities relates to one facet of community behavioral health, the local crime rates and the perception of safety in these communities.

### 1.1 Social Organization: Collective Efficacy and Social Trust

Across the literature a number of different characteristics have been included under the umbrella of social organization, including collective efficacy, social trust, attachment, and (informal) social control, organizational participation, local friendship networks, and problematic adolescent groups, as well as broader concepts like social capital, to name a few (Bjornstrom, 2011c; Osgood, 2000; R. D. Putnam, 1995; Sampson, 1997; Shaw & McKay, 1999; Wilkinson, Kawachi, & Kennedy, 1998) Kaylen & Pridemore, 2013. The current study focuses on two central components of social organization – social trust and collective efficacy – that have been consistently related to crime, delinquency, and perceived crime in urban or mixed urban and rural samples (Elliott et al., 1996; Kawachi, Kennedy, Lochner, & Prothrow-Stith, 1997; Sampson, 1997; Sampson, Morenoff, & Earls, 1999; Wilkinson et al., 1998; Xu, Fiedler, & Flaming, 2005).

Towards this end, we are particularly interested in investigating the predictors of social organization and crime: poverty, income inequality, ethnic or racial heterogeneity, and mobility (Kawachi et al., 1999; Kennedy, Kawachi, Prothrow-Stith, Lochner, & Gupta, 1998; Lowenkamp, Cullen, & Pratt, 2003; Osgood & Chambers, 2000; Sampson, 1997; Shaw & McKay, 1999). The full social disorganization model presupposes that high levels of certain structural characteristics such as poverty, mobility, ethnic or racial heterogeneity, and income inequality are posited as barriers to communication, cohesion, and creating shared values, thereby producing low levels of social organization among community members. Low levels of social organization are proposed to lead to high levels of crime and perceived crime. Additionally, these factors may create feelings of division and alienation among community members. Rather than promoting the feeling of togetherness, these structural factors likely undergird the development of distrust – a quality that has been identified both as a contributing factor to, and a consequence of, crime (Galea, Karpati, & Kennedy, 2002).

This study focuses on the role of collective efficacy and social trust. Collective efficacy includes elements of social cohesion and social control and is defined as the degree to which community residents work together to achieve shared values and solve community problems (Bjornstrom, 2011a; Sampson, 1997; Sampson, Raudenbush, & Earls, 1997). Therefore, even though there are elements of cohesion within shared values, collective efficacy's emphasis seems to focus on action. On the other hand, social trust can be defined as believing that others have a general desire to do good (Kennedy et al., 1998). Social trust, then, seems to be more about personal attitudes and beliefs, and does not necessitate cohesion or action. It is generally thought that social trust is a *precursor* to collective action and/or cooperation which, in turn, reinforces positive feelings of social trust (Flanagan & Stout, 2010). Given the different foci of the two concepts, collective efficacy as cohesion and action whereas social trust as belief in the goodwill of others, it is possible that they could relate differently to measures of crime.

This work has been largely focused on urban areas, leaving the question of whether these associations generalize from studies of mostly urban communities to rural communities and small towns. Important to note, one recent prior study with rural areas suggests that this full social disorganization model does not generalize to rural communities (Kaylen & Pridemore, 2013). Further, these two constructs may be predicted by different characteristics when the context changes from urban or mixed urban and rural samples to a focus on rural and small town communities.

### 1.2 The Rural Context

Intellectual effort to understand the impact of *place*, including its social organization, on human growth and development, has flourished over the past decade. Yet, research on the social organization of rural communities remains fairly limited. A few studies have previously examined the association between structural community characteristics and archival crime rates; however, the findings have been inconsistent. For example, one study found poverty to be unrelated to crime, and income inequality strongly related to *some* crimes (Deller & Deller, 2010). Poverty and unemployment have not significantly related to juvenile crime rates (Osgood & Chambers, 2000), and levels of disadvantage and mobility were not significant predictors of juvenile violence measured by violent victimization rates recorded by hospitals (Maria T. Kaylen & Pridemore, 2011).

Yet, other research in rural samples has demonstrated the opposite. Specifically, unemployment and minority concentration were strong predictors of crime in one study (Deller & Deller, 2010) and a few studies have shown that community disadvantage significantly predicted crime (Barnett & Mencken, 2002; Chilenski & Greenberg, 2009; Lee & Ousey, 2001; Lee & Thomas, 2010). Ethnic heterogeneity, residential instability, and measures of family disruption have also significantly predicted juvenile crime in rural areas (Osgood & Chambers, 2000).

The limited research on perceived social organization has also shown mixed results. For example, Chilenski and Greenberg (Chilenski & Greenberg, 2009) found no association between collective efficacy and general crime rates, rates of adolescent alcohol use, aggressive behavior, or property destruction, but did find a relationship with adolescent cigarette use. In contrast, Reisig and Cancino (Reisig & Cancino, 2004) reported a significant negative association between collective efficacy and burglary. Still, other studies suggest that social cohesion may be more salient than informal social control in rural areas. Reisig and Cancino (Reisig & Cancino, 2004), for example, found that social cohesion had a stronger relationship with perceived crime and burglary; while another study found that neighborhood disadvantage was negatively associated with youth reports of social cohesion in rural areas (Witherspoon & Ennett, 2011). Most recently, one study demonstrated a lack of association among community structural characteristics, measures of social organization, and measures of crime in rural areas (Kaylen & Pridemore, 2013). Given the inconsistent nature of associations, it is possible that there are other important factors that relate to crime in rural areas. Consequently, the current study adds a measure of perceived safety while attempting to replicate the full social disorganization model in rural communities.

## 2.0 The Current Study

Here we examine how social trust and collective efficacy relate to community archival crime rates and perceived community safety in rural and small town communities in two states. Given the lack of consensus in the extant research of how these social organization characteristics relate in rural areas, we examine a series of competing hypotheses in order to better understand these associations. Community rates of poverty, income inequality, mobility, and racial composition are included in order to assess the ways that structural characteristics shape social organization and crime.

**Figure 1:**
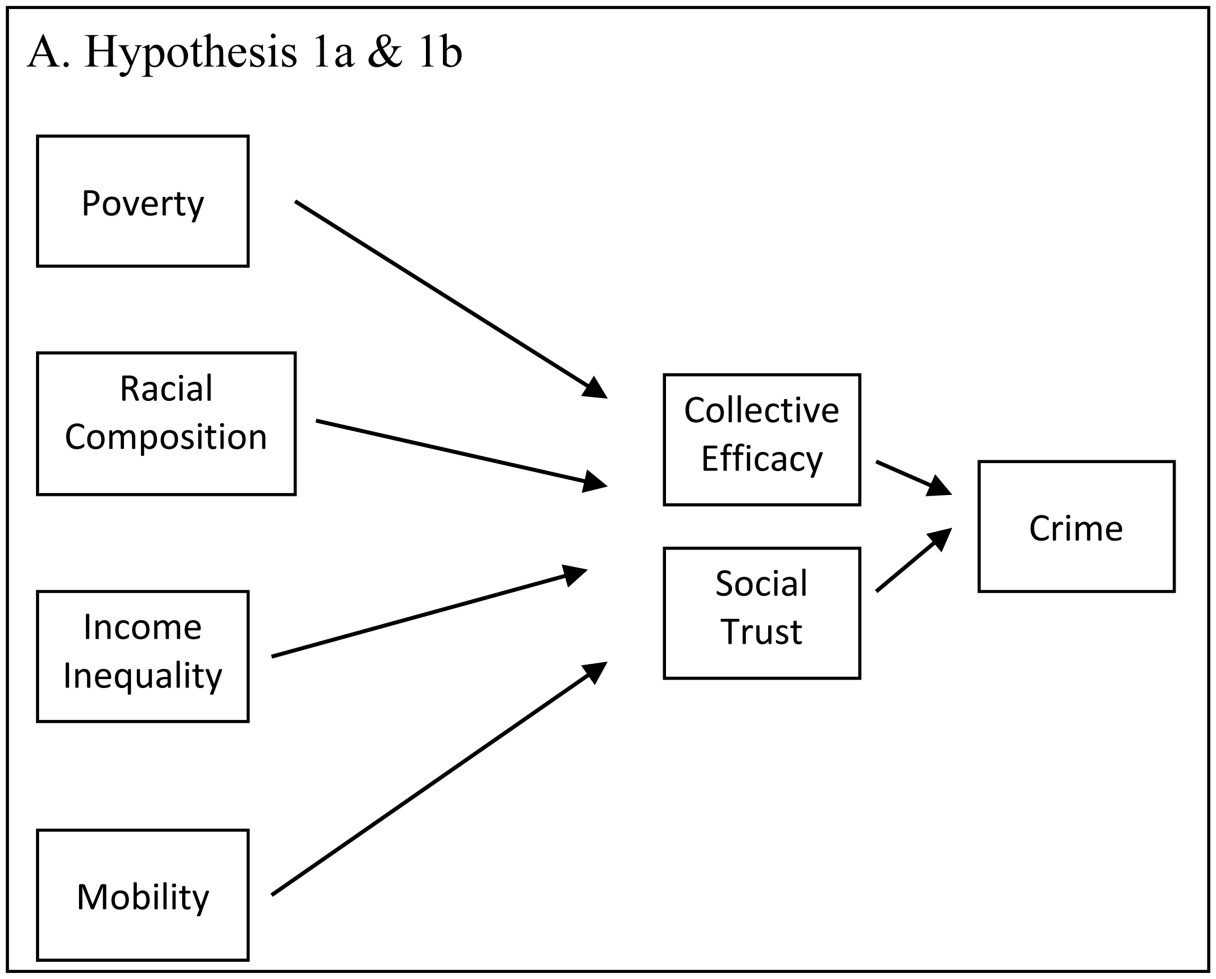

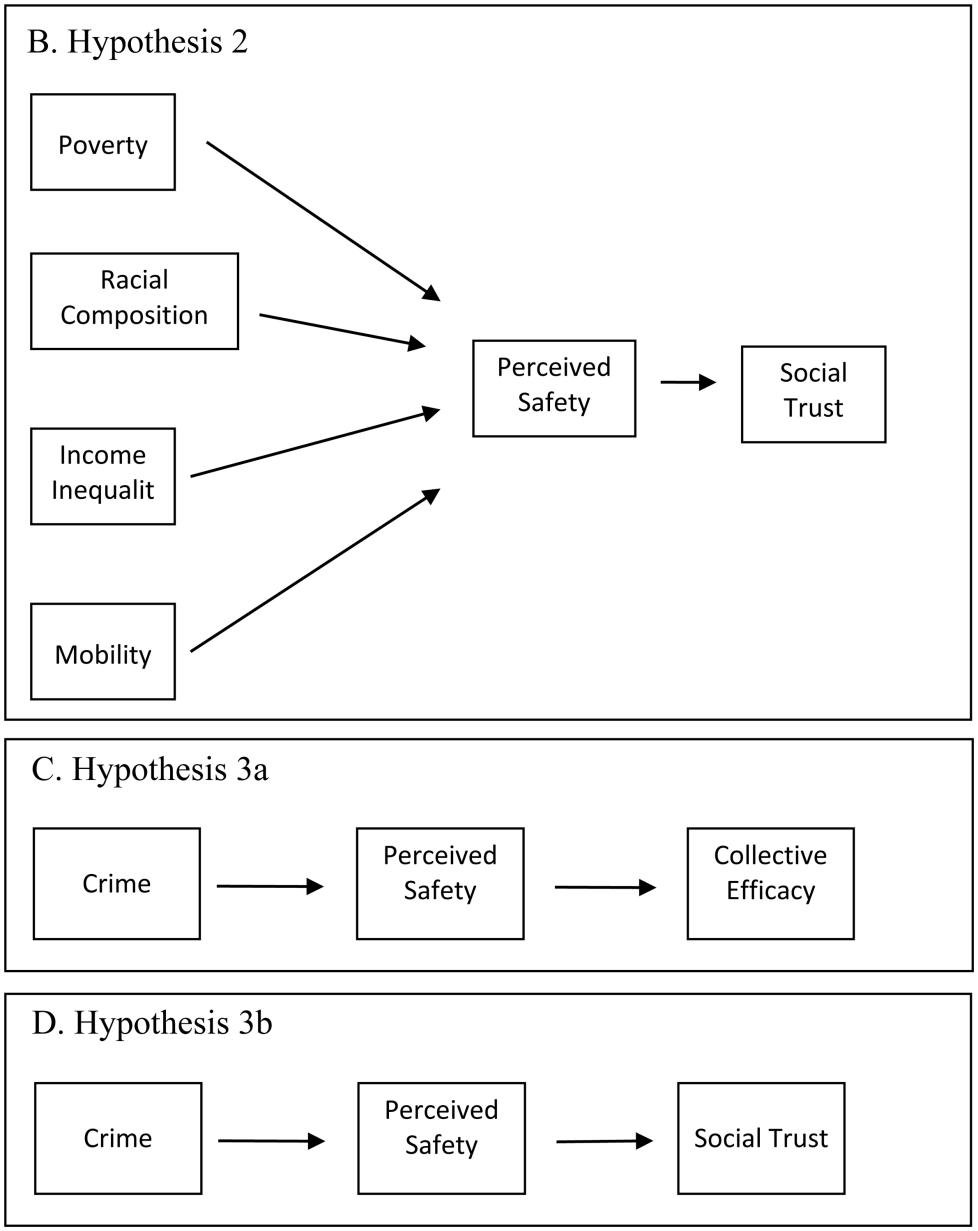
Hypothesized associations between structural characteristics, social organization, crime, and perceived safety

The analyses were conducted in three steps. First, we tested whether collective efficacy mediates the relationship between poverty, income inequality, mobility, and racial composition, with crime, or whether social trust mediates those relationships (see Figure 1a). Second, we tested whether perceived safety was a mediator between community structural characteristics and social trust (see Figure 1b). Third, we tested whether perceived safety mediates the relationship between crime and collective efficacy (see Figure 1c), or whether perceived safety mediates the relationship between crime and social trust (see Figure 1d).

## 3.0 Method

The data for this study come from the 27 rural and small town school districts involved in the PROSPER Project (*PRO*moting *S*chool/community-university *P*artnerships to *E*nhance *R*esilience (Spoth, Greenberg, Bierman, & Redmond, 2004). Tested in Iowa and Pennsylvania, the PROSPER Project utilized a randomized control design which created local community prevention teams with the goal of implementing evidence-based programs to support positive youth development and reduce early substance use. Primary eligibility criteria for communities were that school districts needed: (a) to be located in non-metropolitan areas and enroll between 1,301 to 5,200 students in grades K-12, (b) to have at least 15% of its families qualify for free or reduced-price lunches, (c) to have less than half of the population employed by or attending a university, and (d) not to be involved in other university-affiliated youth prevention research. University Institutional Review Boards approved the study before participant recruitment began.

### 3.1 Participants and Procedures

These analyses draw from two distinct data sources within the PROSPER Project: key community member data and archival community structure data. Key community members were invited to complete an annual survey about the health, well-being, and characteristics of youth and families in their communities. The analyses draw on the data collected in Fall 2006, the wave that included the most intensive measurement of community social organization. The archival structural data comes from multiple sources (see Table 1).

Two-hundred and twenty-six individuals participated in the semi-structured interviews in the fall of 2006. In the intervention communities, key community members included the team members on each local prevention team: local stakeholders from Cooperative Extension, the school, local mental health and substance abuse agencies, and parents. In the control communities, similar community representatives participated. Participants ranged in age from 22 to 62 (*M* = 43, *SD* = 8.9), 69% were female, and 99% self-identified as White. Most participants had a college degree (92%) and most (80%) lived in or near the community on which they reported data. The number of key community members reporting on a given community ranged from 3 to 13 (Median = 6). Collecting information about the community context from a selected group of community representatives rather than a census or random sample is quite common within community intervention research (Brown, Hawkins, Arthur, Abbott, & Van Horn, 2008; Javdani & Allen, 2011). It facilitates efficient data collection from multiple geographically distinct communities, yet it is possible that there are different dynamics of how these key community members observe, experience, and represent their communities compared to a random sample or census sample.

### 3.2 Measures

#### Economic risk

Two indicators were used to gauge community-level economic risk: the percent of families in the school district that live below the US poverty threshold (National Center for Education Statistics, 2003) and the percent of students receiving free or reduced-cost lunches as reported by the school district. These measures were standardized and averaged (*M* = 0, *SD* = 1.0).

#### Income inequality

US Census data was used to calculate the Gini coefficient (National Center for Education Statistics, 2003). The Gini coefficient is among the most commonly utilized measures of income inequality (Kawachi & Kennedy, 1997). Complete equality (i.e., income is shared equally among all households) results in a Gini coefficient of zero. Conversely, complete inequality (i.e., only one household has all the income and the rest have none) results in a Gini coefficient of 1.00 (see (Nielsen, 2006) for additional details). For these analyses, the Gini coefficient was transformed by multiplying it by 100.

#### Racial heterogeneity

Racial heterogeneity was measured with US Census data as the percentage of the school district population who self-identified as being only White (National Center for Education Statistics, 2003). White includes persons having origins in any of the original peoples in Europe, the Middle East, or North America. For ease of interpretation, this variable will be referred to as *percent White* in the proceeding text.

#### Mobility

The percent of the total school district population (over age 5) that resided in a different house in 2000 than they did in 1995 was derived from data calculated from US Census (National Center for Education Statistics, 2003).

#### Crime

Drawing from the FBI's Uniform Crime Reporting System, violent, property, and narcotic crimes were aggregated over a three-year period (2002-2004) to compute an estimated yearly rate of crime (Coco, 2005; Pennsylvania State Police, 2005). To compute the estimated number of crime incidents within each school district, each crime jurisdiction's reported number of incidents (not county aggregates) was weighted by the percent of their geographic area that was contained within the school district boundaries, summed, and then multiplied by 100,000 to create a rate of crime incidents per 100,000 residents. See Chilenski and Greenberg (2009) for additional detail about this measure. The crime index was standardized (*M* = 0, *SD* = 1.0).

#### Collective efficacy

Two subscales from the key community member interviews were averaged to assess collective efficacy: community attachment and community initiative (Chilenski & Greenberg, 2009). The community attachment scale (3-items; α = .56) is conceptually equivalent to the cohesion component of collective efficacy, by measuring the level of resident investment and closeness in the community with the following three items: All things considered, most people are satisfied with this community as a place to live / Most people care greatly about what this community is like / Most people who live here feel a strong tie to the community. The community initiative scale (4-items; α = .65) is conceptually equivalent to the informal social control component of collective efficacy, by gauging the perceived extent of citizens' active engagement in the community with the following four items: It is difficult to get people in this community involved in community activities (*reversed*) / most people in this community are committed to addressing community isues / this community is willing to try new ideas to solve community problems / most people in this community are pretty set in their ways. All seven items were measured on a 4-point scale, from *Strongly Disagree* (1) to *Strongly Agree* (4).

#### Social trust

Three classic indictors of social trust (Galea et al., 2002) were revised to assess key community members' perceptions of citizens' generalized belief that humanity is fair, trustworthy, and helpful (α = .74). Items included: (a) In this community, people generally believe that most people can be trusted; (b) In this community, people think most people are fair; and, (c) In this community, people generally believe that other people just look out for themselves, rather than try to help others (reverse coded). All items were scored on a 4-point scale where 1 = *Strong Disagree* and 4 = *Strongly Agree*.

#### Perceived safety

Key community members responded to 6 questions (α = .77) that were revised to assess perceptions that community members feel safe in their community (Robinson, Lawton, Taylor, & Perkins, 2003). The items assessed multiple aspects of safety including felt safety when out alone (during the day and at night) or when coming to the school for meetings/programs, as well as a more generalized safety measure gauging the belief that this community is a safe place to live. All items were scored on a 5-point scale: 1 = *Strongly Disagree*, 3 = *Neutral*, 5 = *Strongly Agree*.

### 3.3 Analysis Plan

The data were analyzed in several steps. First, preliminary analyses examined bivariate correlations to gauge the associations between community-level variables and individual-level variables. Due to the experimental design and the timing of data collection, we examine differences by state and experimental status. Second, the three sets of competing hypotheses were tested using mediation analysis (PROC REG and PROC MIXED in SAS 9.1). Multilevel mediation models were used to test Level 1 mediation of a Level 2 effect when the independent variable(s) was at the community-level (Level 2) and the mediator and dependent variable were at the individual-level (Level 1) (Krull & MacKinnon, 2001). Drawing from Baron & Kenny (Baron & Kenny, 1986), the following process was used to test each hypothesis: (a) Model 1: Dependent variable was regressed on the independent variable(s) (X → Y); (b) Model 2: Mediator was regressed on the independent variable(s) (X → M); (c) Model 3: Dependent variable was regressed on the independent variable(s) *and* the mediator (X + M → Y). After estimates for all four models were computed, the Sobel test (Preacher & Leonardelli, 2012) was calculated to test the significance of the mediation (e.g., the significance of the indirect effect).

## 4.0 Results

### 4.1 Preliminary Analyses

Descriptive statistics are located in Table 1. Generally, sample communities have low rates of poverty and mobility and are limited in racial-ethnic diversity. Both crime rates and rates of income inequality are heterogeneous.

#### Community structural characteristics

Bivariate correlations are displayed in Table 2. Correlations of structural characteristics reveal that crime has a positive, moderate association with economic risk (*p* = .02) and mobility (*p* = .02), and a negative, moderate association with percent White (*p* = .02). That is, communities with higher crime rates had significantly higher levels of economic risk, higher rates of mobility, and a more ethnically heterogeneous population. Mobility had a strong, negative association with percent White (*p* < .0001); that is, communities with higher rates of mobility were more ethnically heterogeneous. Income inequality was not significantly associated with any structural characteristics.

#### Social organization characteristics

Correlations among the three social organization variables (perceived safety, trust, and collective efficacy) revealed moderate positive associations (*p* < .0001; see Table 2). These associations were also significant at the community-level; correlations of the community-level averages of the three social organization variables were all moderate to strong and positive.

#### Community structural characteristics with social organization characteristics

Only one social organization characteristic was significantly associated with the community structural characteristics: perceived safety had a small, positive association with percent White (*p* = .07) and a moderate to strong, negative association with crime rates (*p* = .01). In other words, communities perceived as safer were less ethnically heterogeneous and had lower crime rates.

#### Associations with state and experimental status

State of residence and experimental status were associated with select structural and social organization characteristics. Compared to Iowa communities, there are lower rates of mobility (*r* = -.67, *p* < .001), crime (*r* = -.37, *p* = .06), and collective efficacy (*r* = -.43, *p* = .02) in the Pennsylvania communities. Communities randomly assigned to the control condition reported lower social trust (*r* = -.36, *p* = .06). As a result, state and experimental status were included as controls in the final models.

### 4.2 Competing Mediation Hypotheses

#### Does collective efficacy or social trust mediate the association between structural characteristics and crime?

The first set of models tested competing hypotheses about the roles collective efficacy (H1_A_) and social trust (H1_B_) play in mediating the association between community structural characteristics and crime. Because crime is at Level 2 (i.e., community-level), these models were conducted with OLS hierarchical regressions using proc reg in SAS version 9.1 and our sample size is 27. (see Table 3). Tests of H1_A_ revealed that economic risk (*p* = .02) and mobility (*p* = .07) were the strongest predictors of community-level crime. Tests of the indirect effect reveal that collective efficacy did not mediate the association between structural characteristics and crime. The results for H1_B_, in which social trust replaced collective efficacy as the mediator, followed a similar pattern: economic risk (*p* = .02) and mobility (*p* = .07) were the strongest predictors and social trust did not mediate the association between structural characteristics and crime.

#### Perceived safety as a mediator between structural characteristics and social trust

Because the dependent variable is a Level 1 (i.e., individual-level reported) variable, and we have predictors at both Levels 1 and 2, these analyses were conducted as multi-level mixed models using proc mixed in SAS, Version 9.1. The model for H2 assesses whether the association between community structural characteristics and social trust is mediated by perceived safety. As shown in Table 3, the strongest predictor of social trust is perceived safety (*p* < .0001). None of the structural characteristics are significantly related to either perceived safety or social trust. Tests of the indirect effect reveal that perceived safety does not mediate the association between structural characteristics and social trust. The standardized total effect size was calculated to help with interpretation of the two significant models. The standardized total effect for crime to collective efficacy, via perceived safety was -.10. The standardized total effect for crime to social trust, via perceived safety was -.23.

#### Perceived safety as a mediator predicting collective efficacy or social trust

The next set of hypothesis tests (H3_A-B_) examined whether perceived safety mediates the association between crime and collective efficacy, or whether perceived safety mediates the association between crime and social trust. Because the dependent variable is a Level 1 (i.e., individual-level reported) variable, and we have predictors at both Levels 1 and 2, these analyses were conducted as multi-level mixed models using proc mixed in SAS, Version 9.1 (see Table 3). Tests of H3_A_ indicate a strong, negative association between crime and perceived safety (*p* = .003), and a strong, positive association between perceived safety and collective efficacy (*p* = .01). Tests of the indirect effect reveal that perceived safety partially mediated the association between crime and collective efficacy (*p* = .05). Results from H3_B_ also indicate a strong, negative association between crime and perceived safety (*p* = .003), and a strong, positive association between perceived safety and social trust (*p* < .0001). Tests of the indirect effect reveal that perceived safety partially mediated the association between crime and social trust (*p* = .005).

## 5.0 Discussion

To date, most research regarding social organization has been conducted in urban settings, leaving the question of how these social organization domains are related to community structural characteristics in rural and small town communities. The goal of the current study was to examine how collective efficacy, social trust, and perceived community safety relate to one another as well as to a range of community-level structural characteristics and crime. Results from bivariate and mediation analyses indicate that the full theorized social disorganization model that has been tested in urban areas is not generalizable to rural and small town communities. These results replicate one other recent study of this model in rural areas (M. T. Kaylen & Pridemore, 2013). More specifically, correlation analyses revealed that strong predictors of crime in these rural areas were economic risk, mobility, and ethnic heterogeneity, yet, collective efficacy and social trust did not mediate these associations when combined in multivariate models. Additional analyses revealed that perceived safety may play an important role in the social organization of rural communities. It demonstrated a significant link between crime and social trust, and a significant link between crime and collective efficacy, in rural and small town communities. Understanding the relationships among these constructs in rural and small town communities will help inform community prevention efforts and policies targeted to improving social organization in these areas.

### 5.1 Understanding Crime in Rural and Small Town Communities

This is one study to add to the body of literature focused on understanding the impact of place on human growth and development. Some of the same social disorganization constructs that are important predictors of crime or juvenile delinquency in urban areas also important in rural and small town communities, but the full social disorganization model which includes measures of residential involvement and attachment such as collective efficacy (Sampson, 1997), does not generalize to rural and small town communities. This study replicates other research on social disorganization in rural areas that used different intervening measures of social organization and survey-measured crime dependent variables (M. T. Kaylen & Pridemore, 2013), further strengthening the conclusion that social organization in rural communities operates differently than it does in urban communities.

Specifically, economic risk was the strongest predictor of crime in rural and small town communities in multivariate analyses; mobility and ethnic heterogeneity only significantly related to crime in correlation models, and collective efficacy and social trust did not directly predict crime. The significant association between economic risk and crime was also found in other research on rural areas (Barnett & Mencken, 2002; Lee & Ousey, 2001; Lee & Thomas, 2010); and the association between mobility and crime and ethnic heterogeneity and crime has been found in a study using rates of juvenile delinquency as the measure of crime (Osgood, 2000). These relationships seem to be quite important, whether they have a direct or indirect association with crime.

### 5.2 How Rural Communities and Small Towns are Different

There must be differences in how social organization characteristics are related to crime in rural and small town communities that are explained by differences between rural and urban communities. Prior research has suggested a range of factors such as rural communities have: (a) less dense residential areas, (b) selective out-migration of highly educated young people, (c) higher mean levels of social organization, and (d) the likelihood of law enforcement to make an arrest when a crime is committed (Kaylen & Pridemore, 2013; Ceccato & Dolmen, 2011; Lee, 2008).

We propose some additional possibilities. There may be a lack of anonymity in rural and small town areas, which may make it less likely for crimes to be committed regardless of the levels of social organization. A possible perpetrator may be more likely to believe he or she will be arrested when greater levels of anonymity exist. On the contrary, there may also be a stronger value and expectation of privacy in rural areas, which may make it more likely for individuals to commit a crime regardless of the levels of social organization; a potential perpetrator may expect possible witnesses to keep to themselves or even not to notice what is happening in the environment outside of their home. In extremely rural areas, there may even be an absence of witnesses due to the larger distance between residences. Alternatively, families may be less mobile and family members may tend to live in the same town at a greater occurrence than in urban neighborhoods, which may increase levels of social trust which have nothing to do with crime levels. Increased levels of residential stability may make it likely for reputations of families or certain family members to be passed down from older generations, making it less likely for someone to intervene when there is a problem. These factors may make it less likely for residents to believe that positive change can occur, regardless of crime rates.

Crimes may be reported less frequently in rural areas compared to urban areas. Community members in rural areas and small towns may have alternative techniques they employ when individuals break the law. All of these factors, and even others could change the association among the various social organization characteristics, and change associations between social organization and crime. Future research in rural communities needs to more completely examine the potential unique mechanisms that create social organization and affect individual and community health, and specifically crime, in these areas.

### 5.3 Perceived Safety in Rural Communities

The current study extended traditional social disorganization research by including a measure of perceived safety. Results from multi-level mediation analyses demonstrate that perceived safety was a significant link between crime and social trust, and crime and collective efficacy. Specifically, community levels of crime predicted perceived safety, and then levels of perceived safety predicted collective efficacy and social trust. Unlike previous research conducted in urban or mixed urban and rural areas (Kawachi et al., 1999; Sampson, 1997; Sampson & Graif, 2009; Wilkinson et al., 1998), the direct association between crime and collective efficacy, and crime and social trust was not significant in any of the tested models. These results replicate the significant collective efficacy and perceived safety association found by others researching rural or urban communities (Reisig & Cancino, 2004; Sampson & Graif, 2009; Sampson et al., 1997).

Furthermore, these results suggest that collective efficacy is not a predictor of crime in rural and small town communities, but a community's level of collective efficacy may be affected by residents' perceptions of safety which are affected by crime rates. Longitudinal research is needed to test this causal pathway. These results have several possible implications for interventions in rural and small town communities. First, these results suggest that some communities with similar levels of crime may perceive the level of safety in their community differently. Additionally, because collective efficacy and social trust did not predict crime in these rural areas, interventions that are aimed to improve levels of collective efficacy and/or social trust in a community may be unlikely to affect crime rates. Though the structure of this data collection limits our ability to make causal conclusions, this finding deserves attention. Isolating the linkages among these variables will make it more likely that community efforts at reducing crime will be targeted at the appropriate pathways. In other words, these results suggest that community efforts aimed at improving collective efficacy, even if successful, may be ineffective at reducing overall crime and juvenile delinquency.

Crime is only a moderate predictor of perceived safety indicating that there are other mechanisms that help residents feel safe. In other words, there is some variability in levels of perceived safety even when communities have similar levels of crime. Additionally, these analyses suggest that perceived safety may be a precursor to building collective efficacy and social trust. It is possible, then, that without building or addressing perceived safety, efforts aimed at building social trust and/or collective efficacy in rural and small town areas may not be effective. For example, a study of an urban city demonstrated that perceived disorder and community policing were more important for predicting citizen fear than objectively measured crime rates (Xu et al., 2005).

### 5.4 Implications for Community Policy and Practice

This is one study that can help build a knowledge base to inform practice and policy in rural areas. Our analyses suggest that it is the variation in perceived safety that is unexplained by crime that predicts how people feel about their communities, specifically, how much social trust and collective efficacy are present. It is likely that community leaders and members, the media, and even the police department all play a role in how the general public views criminal acts, however, these factors were unexplored here. Community leaders and members, the media, and police departments that stress the occurrence of the crime incidents with headline news stories may unintentionally make community members feel less safe, which may make them less likely to trust their neighbors and other community residents that they encounter on a daily basis. Such findings may also make them less likely to get involved in an effort to improve their community. As prior research in mixed urban and rural areas suggest, the relationship among these variables may be cyclical and reinforcing; communities that have residents that are less trusting of one another and less likely to get involved in a positive way in their community may then continue to experience high(er) crime rates. Community policing may be one way the linkages among these variables can be addressed (Xu et al., 2005). Less glorified media coverage of crime events and more media coverage of police investigations and judicial success stories may also be helpful. Lastly, these results suggest it may be important to address the levels of perceived safety *before* trying to change levels of social trust and/or collective efficacy in rural and small town communities. Without addressing perceived safety, it is possible that efforts aimed at improving collective efficacy and/or social trust will be ineffective.

### 5.5 Limitations

Inferences from our study findings are limited by our use of a convenience sample. These data were collected as part of an ongoing two-state community-level intervention project, whose original project aims did not include a complete examination of the community context. Additionally, respondents were selected key community members, rather than a representative sample of community residents. Because of how these communities were selected, it is not likely to be representative of *all* rural areas across the US; rather, these communities are relatively representative of rural and small town communities in the Northeastern and Midwestern United States (Chilenski & Greenberg, 2009).

The causal inferences that can be made from these findings are limited by the non-longitudinal nature of the studied data. Nonetheless, mediation analyses and/or cross-sectional regression-based analyses have been consistently used with cross sectional and/or non-longitudinal data to investigate associations among constructs in this and other areas of research (Bjornstrom, 2011c; Burchfield, 2009; Comstock et al., 2010; Dassopoulos & Monnat, 2011; Sharp, Caldwell, Graham, & Ridenour, 2006). It is possible that the directional pathways between these constructs function in the opposite way than we investigated them, however, we tried to focus on directional pathways that were theoretically likely. For instance, perceived safety is not likely to be a predictor of crime; it seems more likely that perceptions of safety/crime are the results of actual crime events, rather than contributors to actual crime events. In addition, though these data are not longitudinal, they were also not all collected at the same time and covering the exact same years. Planned longitudinal data of all constructs would provide a stronger examination of this research idea.

This study used a unique approach to measure crime in nontraditional but meaningful crime jurisdictional areas, school districts (Chilenski & Greenberg, 2009). Each school district was typically composed of multiple smaller crime jurisdictional areas, such as unique municipalities or towns, and some unincorporated areas that were under the jurisdiction of a county or state police department. This method produced crime rate estimates that were well validated (Chilenski & Greenberg, 2009). Yet, crime does not occur evenly across space and the Uniform Crime Reporting System is flawed. There is likely unknown error within this measure, and not having a premeasured school-district crime index is a potential limitation of the study. Additionally, we did not have a school district measure of female headed households as is commonly used in this area of research.

This is one of the first studies to include multiple social organizational and structural variables in an exclusively rural and small town sample. Therefore, these analyses provide a significant step forward in understanding the importance of the community context in these communities. Results can be generalized to similar rural and small town communities: communities that have relatively low levels of poverty, low levels of mobility, and have relatively low levels of ethnic and/or racial diversity.

Our sample consisted of twenty-seven communities which, while large enough to test our research questions, does pose challenges when it comes to issues of statistical power. In order to protect against Type I error and in light of our small Level 2 *n*, we used a less stringent statistical significance criterion for analyses at Level 2 (i.e., *p* < .10). In reality, this had little effect on our main findings, as they typically met traditional levels. Additionally, this criterion allowed us to interpret some associations at the community-level which mirror significant findings in prior research with larger samples (Kawachi et al., 1997; Osgood & Chambers, 2000; Sampson, 1997)

Some of our Chronbach's Alpha levels were slightly lower than the generally accepted desired level of .70. This is due to creating scales that have a relatively small number of items, a necessity in community intervention research studies. This limitation makes it more difficult to find statistical significance. We used the Sobel test to test for the significance of the indirect effect in our mediation models. There are mixed opinions about using the Sobel test with small samples. Yet, in this area of research, mediation is often tested without the addition of a specific significance test of the indirect effect (Bjornstrom, 2011c; Burchfield, 2009; Comstock et al., 2010). The Sobel test helped us focus on the meaning of the mediating variable; additional research is needed to replicate and quantify these relationships.

## 6.0 Conclusion

The current study examined how collective efficacy and social trust related to multiple measures of community structure and perceived safety. There were three main findings. First, perceived safety was an important link between crime and social trust, and crime and collective efficacy in rural and small town communities, consequently these results suggest that perceived safety may be an important factor in understanding how residents feel about their communities. Second, some of the same factors that predict crime in urban areas predicted crime in these rural communities. Specifically, economic risk, mobility, and ethnic heterogeneity were the strongest predictors of crime in these rural and small town communities. Third, and most important, though some structural variables predicted crime, the full social disorganization model that includes measures of social organization, does not seem to generalize to rural communities and small towns. Longitudinal research is needed to further test these associations; research needs to also include a more detailed analyses of other potentially important mechanisms.

## Figures and Tables

**Table 1:** Community-level descriptive statistics, data source, and time point of data collection for all structural and social organization measures

Measure	Mean	*SD*	Min	Max
*Structural Characteristics*				
Economic Risk				
Community Poverty*	6.81	1.93	1.80	10.70
District Low Income∧	29.45	8.96	10.40	48.00
Income Inequality*	41.97	3.02	35.55	47.05
Percent White*	96.66	3.17	87.80	99.00
Mobility*	37.65	6.15	25.39	45.51
Crime Rates+				
Violent+	284.73	197.35	13.27	674.24
Property+	2,617.19	1,357.17	837.68	6,208.22
Narcotic+	267.28	135.46	82.49	511.34
*Social Organization Characteristics (N = 226)*				
Collective Efficacy#				
Community Attachment#	3.39	.24	2.92	3.86
Community Initiative#	2.49	.25	1.94	2.89
Social Trust#	2.87	.24	2.33	3.33
Perceived Safety#	3.99	.26	3.47	4.53

*Notes. n* = 28 for all Structural Characteristic variables, except for crime rates where *n* = 27; Crime data was averaged across three years, 2002-2004. Averaging crime rates over multiple years is common practice; it supports creating a relatively stable estimate of community crime.

* Census/NCES data source;

∧ School District Reports data source;

+ State Uniform Crime Reports data source;

# Key community member interview data source.

**Table 2:** Zero-order correlations between community-level structural and social organization characteristics

	1.	2.	3.	4.	5.	6.	7.
1. Economic Risk	-----						
2. Income Inequality	.17	-----					
3. Percent White -.12		-.03	-----				
4. Mobility	-.08	-.25	-.61**	-----			
5. Crime Rates	.46*	.12	-.44*	.46*	-----		
6. Collective Efficacy	-.06	.29	-.10	.13	-.02	-----	
7. Social Trust	-.02	-.02	-.13	.14	-.12	.76***	-----
8. Perceived Safety	-.10	.10	.34†	-.16	-.51**	.54**	.63***

*Notes. n* = 28 for all community-level correlations, except for correlations with crime rates where *n* = 27; *n* for Individual-level variables is 226;

† *p* < .10.

* *p* < .05.

** *p* < .01.

*** *p* < .001.

**Table 3:** Mediation Analysis Test of Hypotheses

	Model 1 Total Effect X [c] → Y		Model 2 X [a] → M		Model 3 Direct Effect X [c′] + M [b] → Y		Indirect Effect	
	
	B	*SE*	B	*SE*	B	*SE*	Test Stat.^b^	*SE*
Hypothesis 1_A_: Dependent Variable = Crime								
Economic Risk	.36	.14*	-.03	.05	.35	.15*	0.42	0.03
Income Inequality	.04	.05	.03†	.01	.05	.05	-0.59	0.02
Percent White	-.03	.05	.00	.02	-.03	.05	0.00	0.01
Mobility	.05†	.03	.01	.01	.06†	.03	-0.51	0.01
Collective Efficacy	---	---	---	---	-.43	.72	---	---
Hypothesis 1_B_: Dependent Variable = Crime								
Economic Risk	.36	.14*	-.01	.06	.36	.14*	0.16	0.04
Income Inequality	.04	.05	.00	.02	.04	.05	0.00	0.01
Percent White	-.03	.05	-.01	.02	-.04	.05	0.45	0.01
Mobility	.05	.03†	.00	.01	.05†	.03	0.00	0.01
Social Trust	---	---	---	---	-.61	.60	---	---
Hypothesis 2: Dependent Variable = Social Trust^a^								
Economic Risk	-.01	.05	-.02	.05	.01	.04	-0.40	0.02
Income Inequality	-.01	.02	.01	.02	-.01	.01	0.50	0.01
Percent White	-.01	.02	.03	.02	-.02	.01	1.46	0.01
Mobility	.00	.01	.00	.01	.00	.01	0.00	0.00
Perceived Safety	---	---	---	---	.36	.06***	---	---
Hypothesis 3_A_: Dependent Variable = Collective Efficacy^a^								
Crime	-.03	.05	-.16	.05**	-.01	.05	-1.89*	0.01
Perceived Safety	---		---	---	.14	.06*	---	---
Hypothesis 3_B_: Dependent Variable = Social Trust^a^								
Crime	-.04	.05	-.16	.05**	.01	.04	-2.79**	0.02
Perceived Safety	---		---	---	.34	.06***	---	---

*Notes*. Hypothesis 1a and 1b were tested with Ordinary Least Squares regression; the dependent variable makes the models have an n = 27. Hypotheses 2-3 were conducted as multi-level mixed models using proc mixed in SAS, Version 9.1 (Level 1 n = 226; Level 2 n = 27). B = unstandardized estimates; State of residence and experimental status were controlled in all of the above hypothesis tests. The only significant (marginally) result was for experimental status in H2. Entry of these covariates into the models did not affect the inference of the other associations.

a Model tested a multilevel lower level mediation (individual-level mediator and dependent variable) of an upper level (community-level independent variable) effect.

b The indirect effect test statistic, *p*-value, and standard error were calculated using (Preacher & Leonardelli, 2012).

† *p* <= .10.

* *p* <= .05.

** *p* <= .01.

*** *p* < .001.
